# Parental needs and experiences with e-Health applications in chronic pediatric care – a scoping review

**DOI:** 10.1186/s12887-026-07737-y

**Published:** 2026-09-22

**Authors:** Helena Grüter, Anna Loeffler, Lisa Stähler, Irem Gönen, Jenny Prüfe, Freia De Bock, Claudia R Pischke

**Affiliations:** 1 Department of General Pediatrics, Neonatology and Pediatric Cardiology, Medical Faculty and University Hospital Düsseldorf, Heinrich Heine University Düsseldorf, Düsseldorf, 40225 Germany; 2 Institute of Medical Sociology, Centre for Health and Society, Medical Faculty and University Hospital Düsseldorf, Heinrich Heine University Düsseldorf, Düsseldorf, 40225 Germany; 3 Department of Paediatrics II, University Hospital Essen, University of Duisburg-Essen, Essen, 45147 Germany; 4 Institute for Medical Sociology and Rehabilitation Science, Charité – Universitätsmedizin Berlin, corporate member of Freie Universität Berlin and Humboldt- Universität zu Berlin, Berlin, 10117 Germany

**Keywords:** Chronic condition, Pediatric care, E-Health, Scoping review, Parents, Experiences, Needs

## Abstract

**Background:**

The number of children and adolescents living with a chronic condition is steadily increasing. These conditions often place a considerable burden on affected individuals and their families. E-Health applications have the potential to support families by providing accessible resources and personalized tools for disease management. This scoping review aims to synthesize the specific needs and experiences that parents of children with chronic conditions across different diagnosis categories express in relation to e-Health applications.

**Methods:**

This review was conducted in accordance with the Joanna Briggs Institute (JBI) methodology for scoping reviews and the PRISMA-ScR reporting guidelines. A systematic search of the literature published between January 2010 and February 2025 was carried out in the Web of Science and Ovid databases, including Ovid MEDLINE and APA PsycInfo. The initial search identified 1,173 articles, of which 39 underwent data analysis via narrative synthesis.

**Results:**

A broad spectrum of needs and experiences was identified regarding e-Health applications intended to support parents of children with chronic conditions. Across conditions, such as autism, diabetes, asthma, congenital heart disease, and obesity, recurring themes emerged. Parents consistently emphasized the need for accessible and user-friendly interfaces, reliable and comprehensible health information, and tools to support care coordination and communication with healthcare professionals. Social support and psychological resources provided via e-Health applications were also valued, while concerns regarding data privacy persisted. Experiences with e-Health applications were generally positive, particularly for mobile apps, web-based platforms, and video conferencing, which improved monitoring, empowerment, and access to care. However, parents also reported barriers, including technical difficulties, limited customization, and fear of reduced personal contact with providers. Needs and experiences were frequently repeated across different modalities and conditions, suggesting common priorities for digital health design. Evidence on needs along the care trajectory was limited, with early and long-term phases better represented than transitional periods.

**Conclusion:**

Parents of children with chronic conditions articulate broad, cross-cutting needs for e-Health applications that go beyond diagnosis-specific management. From their perspective, effective tools should integrate accessible design, reliable information, coordination features, social support, and ensure data protection. Research gaps remain regarding transitional care phases and the dynamic evolution of needs over time.

**Supplementary Information:**

The online version contains supplementary material available at https://doi.org/10.1186/s12887-026-07737-y.

## Background

The prevalence of chronic conditions among children and adolescents has been steadily increasing worldwide [1, 2], up to approximately 30% of children are affected globally [3, 4], with quite a range between individual studies. Chronic conditions in childhood encompass a broad spectrum of diseases, such as asthma, attention deficit disorder, or obesity [2, 5], many of which require long-term treatment, continuous medical supervision, and the coordination of multidisciplinary care [3, 6]. A chronic condition in childhood was defined as one that: occurs between the ages of 0–18 years, persists for at least three months or is expected to be long-term, is not curable, or is of a psychiatric nature and demonstrates high resistance to treatment (indicating a limited response to treatment). Alternatively, conditions that occurred at least three times within the past year and were likely to recur were also classified as a chronic condition [2].

These conditions not only impact children’s physical health but also their psychological well-being, social participation, and educational opportunities [3, 7]. The burden of chronic health conditions extends beyond the affected child to parents who are typically required to coordinate care across different providers, navigate complex healthcare systems, monitor symptoms, administer treatment, and acquire medical knowledge [7, 8]. In addition to these organizational and medical responsibilities, parents frequently encounter emotional stress, financial strain, and social stigma associated with their child’s illness [9, 10], partly also decreasing siblings´ wellbeing. This combination of practical and psychosocial challenges for the whole family system underscores the need for comprehensive support structures tailored to families’ diverse needs.

Digital health technologies, including e-Health applications, are increasingly recognized as promising tools to alleviate some of these challenges [11, 12]. E-Health applications are accessible, flexible, location-independent and often cost-effective. They can assist families in tracking symptoms, accessing reliable health information, as well as in managing treatment routines, and communicating with healthcare professionals (HCPs) in a potentially personalized, data-driven way [11, 13]. Moreover, such applications may offer psychosocial support by connecting parents with others in similar situations and providing coping strategies for parents under stress [14].

Much of the existing research on e-Health applications for families has primarily focused on diagnosis-specific e-Health applications, such as those designed for parents of children with asthma, diabetes, and attention deficit disorder [15–19]. While these targeted solutions are valuable, they may not adequately address the broader, cross-cutting needs that many parents share, regardless of their child’s diagnosis. Requiring emotional support, navigating healthcare systems, managing uncertainty, and coordinating care are common across conditions [7, 8]. To date, limited evidence is available on how e-Health applications can address these needs, at what stage throughout the patient journey support via applications is most valuable, and how parents experience using them in everyday life.

This scoping review aims to synthesize evidence on parental perspectives on e-Health applications designed to support management of chronic conditions in children. Specifically, this review addresses the research question: what needs and experiences do parents of children aged 0–16 with a chronic condition express regarding the use of e-Health applications to support and improve the patient journey? The goal is to focus on cross-diagnosis perspectives, highlighting emotional, psychological, social, informational, and organizational dimensions of parental needs and experiences. A further objective is to identify how these needs and experiences evolve across the child’s care trajectory. Finally, the review seeks to identify gaps in the current evidence base in order to inform the design and implementation of future e-Health applications that more effectively support families in managing the complex realities of childhood chronic conditions.

## Methods

This study is a scoping review focusing on the needs and experiences of parents of children and adolescents aged 0–16 years with a chronic condition, including mental health conditions [2], regarding e-Health applications. It was conducted in accordance with the Joanna Briggs Institute (JBI) methodology for scoping reviews [20–22] and reported following the Preferred Reporting Items for Systematic reviews and Meta-Analyses extension for Scoping Reviews (PRISMA-ScR) checklist [23]. Both, study protocol and search strategy, were published in advance on Open Science Framework (OSF, 25.03.2025) [24] to ensure transparency and methodological rigor.

### Search strategy and information sources

Published literature between January 2010 and February 2025 was identified through the electronic databases Web of Science and Ovid, including Ovid Medline and APA PsycInfo. The time span of the searched literature reflects the rapid technological development of digital health tools over the past 15 years [25]. A combination of subject headings and keywords was developed to maximize the specificity of the search. The final search strategy covered five core concepts: (1) chronic disease, (2) parent, (3) child, (4) needs assessment, and (5) e-Health combined through Boolean operators. A comprehensive description of the search strategy has been published elsewhere [24]. In line with the JBI Scoping Review Methodology, broad search terms were applied to ensure the inclusion of diverse relevant articles and a wide spectrum of findings. The search strategy was piloted and then executed once to capture the relevant literature for this review.

### Eligibility criteria

The search was limited to peer-reviewed studies in English and/or German. To gain a broad overview of the research field, all types of study designs were included, for example systematic and scoping reviews. We excluded conference abstracts, published dissertations, and other forms of grey literature as these often lack sufficient methodological detail and peer review.

The population of interest consisted of parents (including primary caregivers and legal guardians) who are responsible for managing the health care needs of a child and/or adolescent(s) aged 0–16 years with a chronic condition [2]. For the selection of chronic conditions, the ICD-10 diagnostic classification (International Classification of Diseases, 10. Revision, German Modification (ICD-10-GM)) was used as a reference. Conditions were included if they met the above-mentioned definition of chronic conditions according to Mokkink et al. [2]. Oncological diseases were excluded from this review, as they are not considered chronic conditions in the narrower sense due to high chances of recovery and because parents face distinct challenges in this context; however, psychological disorders and developmental delays were included. We excluded studies that focused exclusively on adolescents aged 17 years or older to ensure that parents still fulfilled a managerial caregiving role. Studies were further excluded if the study population was not clearly defined or if it was unclear which population was examined in the study.

The review only included articles that primarily focused on the needs and lived experiences of parents in relation to e-Health applications. The term “e-Health application” is not standardized; in this review, it was defined as any digital tool (e.g., web-based platforms, mobile health apps, telemedicine services) designed to support the management, coordination, or psychosocial aspects of chronic care in children and adolescents [26]. It is important to acknowledge that e-Health applications may differ substantially in their intended use and implementation. In particular, telemedicine-based interventions may involve direct healthcare provider – patient interactions, whereas other digital health tools may primarily support self-management, information provision, monitoring, or communication.

### Study selection

Titles and abstracts (stage 1) and full-text (stage 2) were independently screened against the inclusion and exclusion criteria using the data management platform Covidence. Two researchers (H.G. and I.G.) reviewed each article independently (stage 1 and 2). In case of disagreement, a third reviewer (first L.S., then A.L.) resolved the conflict. The involvement of a third reviewer at each stage minimized bias and improved consistency of decisions.

Before the formal screening process, the inclusion and exclusion criteria were piloted on a subset of studies. After initial disagreement, the research team collaboratively refined the criteria to ensure a shared understanding before proceeding with the full screening. After title and abstract screening, full-texts were screened again to exclude unintended secondary findings not directly related to the research question. Risk of bias was not assessed, as scoping reviews are intended to map existing evidence rather than evaluate the quality of the included studies [21].

### Data extraction

Data extraction was independently performed by two researchers (H.G. and I.G.), and conflicts were resolved by a third independent researcher (A.L.). A standardized data extraction template was developed and piloted to ensure consistency across reviewers. Extracted data included: study characteristics (title, author, year, country, study design, outcome measure approach, aim, outcomes measured, tools/instruments used, limitations), parent characteristics (number of parents participating, parent age, type of parent (mothers/fathers, both parents)), child characteristics (chronic condition, care pathways stage, child age, sample size), characteristics of e-Health application details (name, purpose, duration & frequency, type, parent involvement), study results (parent experiences, needs). Inter-rater reliability was assessed for both stages, yielding κ = 0.50 (87.6%) for title/abstract screening and κ = 0.46 (77.1%) for full-text screening, indicating a substantial level of consistency between reviewers. The abbreviation “n.i.” indicated that no information was available, while “n.a.” indicates that the respective variable or item was not applicable.

### Data synthesis

The extracted data were then collated and synthesized thematically to answer the research questions. A narrative synthesis was performed in accordance with the updated JBI methodological guidance for scoping reviews, which involves systematically organizing study findings, developing a preliminary synthesis, exploring patterns and relationships within and across studies, and assessing the robustness of the synthesis. These steps were applied iteratively to summarize and structure the results of the included studies [21].

## Results

### Study selection

**Fig. 1:**
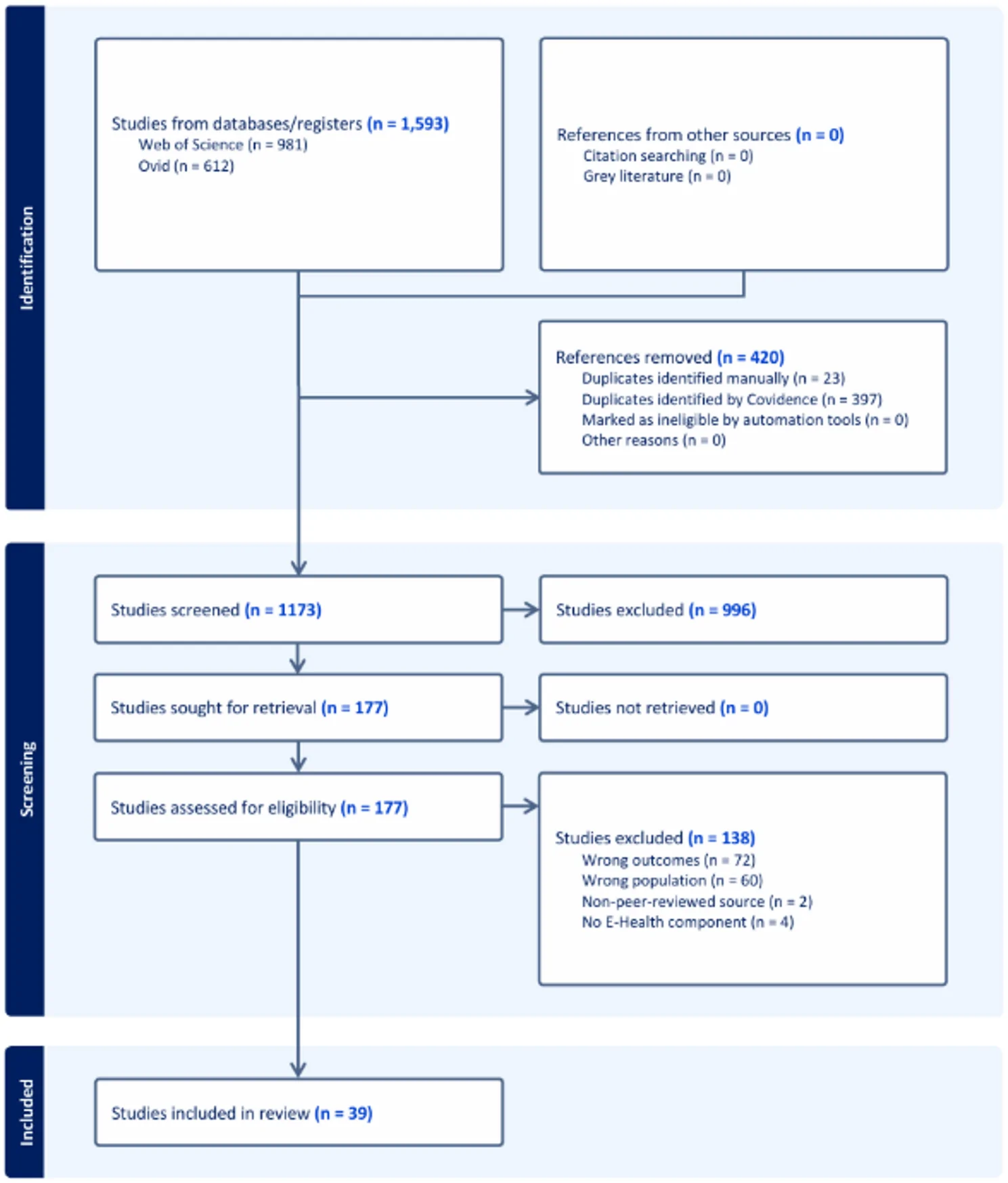
PRISMA flow-chart

The database search identified 1593 records. After 420 duplicate records were removed, 1173 title and abstracts were screened. A total of 39 studies met the eligibility criteria and were included in the review. The complete study-selection process is depicted in Fig. 1.

### Characteristics of the included studies

The included studies were published between January 2010 and February 2025 and were conducted in USA [27–45], Canada [46–50], Australia [51–55], UK [56, 57], Germany [58–60], Norway [61, 62], New Zealand [63] and Sweden [64]. Designs spanned qualitative interviews/focus groups [29–31, 33, 35–38, 42, 43, 45, 47–50, 52, 54, 56–60, 62–65], mixed methods [27, 28, 32, 34, 39–41, 44, 46, 53, 55, 61], case [28, 38] or pilot studies [55] and one literature review [61]. Sample size ranged from 1 to 132 participants. Children in the study ranged in age from 0 to 20 years, and were living with chronic conditions including autism (*n* = 10), diabetes (*n* = 10), congenital or acquired heart disease (*n* = 4), asthma (*n* = 4), or obesity (*n* = 4). The duration, severity, and stage of the children’s condition were reported inconsistently across the studies. A detailed summary of each study’s setting, design, parental sample, chronic condition, care pathway stage and e-Health application is presented in Table 1.

**Table 1:** Summary of included studies

Nr.	Authors, year of publication	Country and study design	Parental sample	Chronic condition	Care pathway stage	e-Health application	
						Type	Purpose
1	Abdulhussein, Pinkney [46], 2023	Canada, web-based survey	99	Type 1 Diabetes (T1D)	n.i.	web portal/web-based programs/platform	Health monitoring and communication with health-care provider
2	Albanese-O’Neill, Schatz [27], 2019	USA, mixed methods	63	T1D	Long-term care / disease management	web portal/web-based programs/platform	Diabetes self-management education and resources support
3	Amsbary, Lin [28], 2022	USA, single-case design	12	Autism spectrum disorder (ASD)	n.i.	web portal/web-based programs/platform	Enhancing social-communication and play skills for young children with ASD
4	Andrews, Nitchie [29], 2019	USA, qualitative	20	Asthma	Periods of high-risk, e.g., transition from hospital to home, ongoing management	Mobile technology	Medication adherence support, risk communication
5	Bowers, Tomlinson [56], 2024	UK, qualitative	4	Congenital heart disease	Time after discharge, long-term care	Mobile apps	Health monitoring, decision support tool
6	Blair, Vergales [30], 2022	USA, qualitative	11	Single ventricle physiology	n.i.	Mobile apps	Home monitoring, family management
7	Bomba, Müller-Godeffroy [58], 2018	Germany, qualitative	9	T1D	n.i.	continuous glucose/health monitoring	Help with management of type 1 diabetes
8	Bui, Pohl [47], 2022	Canada, qualitative (user-centered design approach)	21	Neurodevelopmental disorders	n.i.	Chatbot	Education and overall support for parents
9	Bush, Stahmer [31], 2016	USA, qualitative	9	ASD	n.i.	Electronic health record	Access to health information’s, health monitoring and communication with health-care providers, scheduling appointments
10	Byczkowski, Munafo [32], 2014	USA, mixed methods	126	Cystic fibrosis, diabetes mellitus, juvenile idiopathic arthritis	n.i.	web portal/web-based programs/platform	Access to child´s medical information, communication with healthcare providers
11	Castro, Chougui [48], 2019	Canada, qualitative descriptive study	18	Osteogenesis imperfecta	n.i.	Mobile apps, web portal/web-based programs/platform, messaging (text/SMS), social media, telehealth/video consultations	Supporting parents´ caregiving needs
12	Denusik, Servais [49], 2023	Canada, qualitative	21	ASD	n.i.	Telehealth/video consultations	Supporting parents with strategies to support their child´s communication, play, and social skill development
13	Desai, Wang [33], 2020	USA, user-centered design principles, iterative cycles of design sessions	10	Children with medical complexity	n.i.	Mobile technology	Supporting disease management and care coordination
14	Doerdelmann, Frielitz [59], 2022	Germany, qualitative stand-alone substudy	7	T1D	Newly diagnosed children (diagnosis no more than 6 months ago)	Telehealth/video consultations	Disease-related information’s and promote security in the therapy management of their child
15	Elbalshy, Boucher [63], 2020	New Zealand, qualitative	12	T1D	n.i.	continuous glucose/health monitoring	glucose monitoring
16	Fidjeland and Oen [61], 2022	Norway, integrative Review	n.a.	Obesity	n.a.	Mobile apps, web portal/web-based programs/platform, Telehealth/video consultations, Messaging (text/SMS), social media	Promotion of healthy lifestyle
17	Garner, Thabrew [65], 2023	New Zealand, qualitative	10	T1D, Type 2 Diabetes	More than 6 months after diagnosis	Mobile apps	Well-being app
18	Geryk, Roberts [34], 2016	USA, Formative study, mixed methods	20	Asthma	n.i.	Mobile apps	Adolescent asthma self-management
19	Healy, Marchand [35], 2018	USA, qualitative	13	ASD	n.i.	web portal/web-based programs/platform	Promotion of physical activity for children with ASD
20	Hermaszewska and Sin [57], 2021	UK, qualitative, focus group design	17	ASD	n.i.	Mobile technology	Supporting overall caregiving needs
21	Hjorth-Johansen, Borosund [62], 2023	Norway, qualitative	9	Congenital heart disease	After discharge from hospital/after surgery or after treatment plan was clarified if no surgery was necessary before discharge (so during hospitalization in some cases)	Mobile apps	Decision support tool and support discharge preparations and follow-up at outpatient clinics
22	Jewell, Funk [36], 2025	USA, qualitative	8	T1D	n.i.	telehealth/video consultations	Diabetes management and caregiver psychosocial well-being
23	Kan, Shaunfield [37], 2021	USA, qualitative	20	Asthma	n.i.	Electronic medication monitoring	Adherence to daily preventive asthma medication
24	Kasparian, Lieu [51], 2017	Australia, cross-sectional design	132	Congenital Heart Disease	n.i.	Mobile technology	Providing resources to support care
25	Lee and Messmer [38], 2024	USA, qualitative case study	1	ASD	1 month after diagnosis	telehealth/video consultations	Support child social communication through parent mediation in natural settings and routines
26	Ludlow, March [52], 2024	Australia, qualitative, guided by participatory and co-design practices	16	Anxiety, Depression	n.i.	Mobile apps, web portal/web-based programs/platform	Support with mental health treatment
27	Marker, Monzon [39], 2019	USA, iterative, user-centered	Stage 1: 22, Stage 3: 14	T1D	n.i.	web portal/web-based programs/platform	Education
28	Parsons, Wilson [53], 2019	Australia, exploratory study	24	ASD	n.i.	Mobile apps	Focus on teaching a parent how to teach
29	Perez Ramirez, Ortega [40], 2025	USA, iterative human-centered design process, usability study	7	Obesity	n.i.	web portal/web-based programs/platform	Obesity treatment: engage in physical activity, healthy affordable meals, and community health worker outreach to address psychosocial needs
30	Pickard, Wainer [41], 2016	USA, mixed methods	28	ASD	n.i.	telehealth/video consultations	Teaching parents to promote their child’s social communication
31	Rogerson, Falkmer [54], 2019	Australia, qualitative	17	ASD	n.i.	Mobile apps	Guiding parents of children with ASD through the process of delivering early intense behavioral intervention
32	Sharifi, Dryden [42], 2013	USA, qualitative	31	Overweight, obesity	n.i.	Messaging (text/SMS),	Child behavior change
33	Slater, Schroeder [43], 2024	USA, qualitative	10	ASD	n.i.	Mobile apps	Behavior change
34	Sox, Gribbons [44], 2010	USA, mixed methods	unclear	Hyperactivity, impulsivity, or attention-deficit/hyperactivity disorder	n.i.	web portal/web-based programs/platform	Supporting data entry into a personally controlled health record
35	Stewart, Letourneau [50], 2011	Canada, qualitative	19	Asthma or allergies	n.i.	Mobile technology	Children´s support group
36	Swanson, Duijff [55], 2025	Australia, non-randomized pilot study	12	22q11.2 deletion syndrome	n.i.	Mobile technology	Supporting parental well-being
37	Tanenbaum, Zaharieva [45], 2022	USA, qualitative	16	T1D	30 days of diagnosis	continuous glucose/health monitoring	Health monitoring (constant blood glucose)
38	Thoren, Janson [64], 2021	Sweden, qualitative	14	Obesity	Treatment	web portal/web-based programs/platform	Behavior change, lifestyle change
39	von Sengbusch, Doerdelmann [60], 2021	Germany, qualitative	24	T1D	n.i.	Telehealth/video consultations	Health monitoring

### Characteristics of the e-Health applications

The experiences of parents with e-Health applications were reported in 17 studies [30, 32, 35, 36, 38–40, 43, 45, 49, 53–55, 60, 62–64], six focused on needs [29, 33, 42, 51, 52, 57], and 16 addressed both [27, 28, 31, 34, 37, 41, 44, 46–48, 50, 56, 58, 59, 61, 65]. Nine studies additionally explored parental needs and experiences across the child’s care trajectory [27, 29, 38, 45, 56, 59, 62, 64, 65]. E-Health modalities included mobile apps [30, 34, 43, 48, 52–54, 56, 61, 62, 65], web portals/web-based programs/platforms [27, 28, 32, 35, 39, 40, 44, 46, 48, 52, 61, 64], telehealth/video consultations [36, 38, 41, 48, 49, 59–61], electronic medication monitoring [37], continuous glucose/health monitoring [45, 58, 63], messaging (text/SMS) [42, 48, 61], electronic health record [31], social media [48, 61], mobile technology (i.e., technology-mediated intervention delivery) [29, 33, 50, 51, 55, 57] and chatbots [47]. Table 2 summarizes the characteristics of each e-Health application, including whether (i) it was used to supplement existing care processes, (ii) served as the first point of contact with the care team, (iii) was implemented continuously or for a predefined period, and (iiii) whether parents received formal training before using the application. The code “not applicable” (n.a.) was assigned when a variable fell outside the scope of a study and therefore could not be meaningfully assessed. This applied to studies that did not evaluate the implementation or use of a specific e-Health application, such as studies focusing on user preferences, design requirements, the development of future e-Health applications, or systematic reviews. By contrast, “no information” (n.i.) was used when a study evaluated a e-Health application but did not report sufficient information to assess a specific implementation-related characteristic. Based on these criteria, some studies [33, 51, 52] were coded as not applicable because they focused on user perspectives rather than the implementation or evaluation of a specific e-Health application. Implementation-related characteristics were frequently coded as n.i. because many studies focused primarily on user experiences, usability, or intervention outcomes, while providing limited information on implementation processes or practical aspects of intervention delivery. Data analysis revealed six themes (accessibility and usability, content and information, coordination, consultation with clinicians, psychosocial support, and data protection), which will be addressed in the following section, as well as their timing across the care pathway.

**Table 2:** Implementation characteristics of the included e-Health applications

Nr.	Authors, year of publication	Supplements other care processes? (Yes/No)	First point of interaction with care team? (Yes/No)	Duration of use (on-going vs. fixed-term)	Implementation context/training
1	Abdulhussein, Pinkney [46], 2023	Yes	n.i.	n.a.	n.i.
2	Albanese-O’Neill, Schatz [27], 2019	Yes	n.i.	n.a.	n.i.
3	Amsbary, Lin [28], 2022	Yes	No	n.a.	n.i.
4	Andrews, Nitchie [29], 2019	Yes	Yes	n.a.	n.i.
5	Bowers, Tomlinson [56], 2024	Yes	n.i.	n.a.	n.i.
6	Blair, Vergales [30], 2022	Yes	No	Qualitative interviews occurred following 1–2 months of continuous use to provide feedback for application iteration focused on long-term usability	n.i.
7	Bomba, Müller-Godeffroy [58], 2018	n.i.	No	n.a.	n.i.
8	Bui, Pohl [47], 2022	Yes	No	n.a.	n.i.
9	Bush, Stahmer [31], 2016	Yes	n.i.	n.a.	n.i.
10	Byczkowski, Munafo [32], 2014	Yes	n.i.	n.a.	n.i.
11	Castro, Chougui [48], 2019	n.a.	n.a.	n.a.	n.a.
12	Denusik, Servais [49], 2023	n.i.	n.i.	Participants enrolled either in the full 12-week program or adapted 6-week program	n.i.
13	Desai, Wang [33], 2020	n.a.	n.a.	n.a.	n.a.
14	Doerdelmann, Frielitz [59], 2022	Yes	No	> 1 year	n.i.
15	Elbalshy, Boucher [63], 2020	Yes	No	n.a.	n.i.
16	Fidjeland and Oen [61], 2022	n.a.	n.a.	n.a.	n.a.
17	Garner, Thabrew [65], 2023	Yes	No	n.a.	Parents received link with instructions, no formal training
18	Geryk, Roberts [34], 2016	n.i.	n.i.	1 week	Research assistant demonstrated how to use the two apps and then allowed parents to explore the apps on their own for approximately 10 min.
19	Healy, Marchand [35], 2018	Yes	n.a.	4 weeks	n.i.
20	Hermaszewska and Sin [57], 2021	n.i.	n.i.	n.a.	n.a.
21	Hjorth-Johansen, Borosund [62], 2023	Yes	No	1 month after discharge	Parents received a 10–15-minute introduction of the main features.
22	Jewell, Funk [36], 2025	Yes	n.i.	12 weeks	No formal training
23	Kan, Shaunfield [37], 2021	Yes	n.i.	12 months	n.i.
24	Kasparian, Lieu [51], 2017	n.a.	n.a.	n.a.	n.a.
25	Lee and Messmer [38], 2024	Yes	n.i.	12 weeks	Before intervention began, parent received a video-recording instruction manual, was introduced to interventionist, participated in an initial virtual meeting where the intervention and recording procedures were explained, and later received an intervention manual before weekly sessions started.
26	Ludlow, March [52], 2024	n.i.	n.i.	n.a.	n.i.
27	Marker, Monzon [39], 2019	Yes	No	n.i.	No formal training
28	Parsons, Wilson [53], 2019	Yes	No	3 months	n.i.
29	Perez Ramirez, Ortega [40], 2025	Yes	n.i.	n.i.	n.i.
30	Pickard, Wainer [41], 2016	Yes	No	n.i.	n.i.
31	Rogerson, Falkmer [54], 2019	Yes	No	n.i.	n.i.
32	Sharifi, Dryden [42], 2013	Yes	No	n.a.	n.i.
33	Slater, Schroeder [43], 2024	Yes	No	3 months	n.i.
34	Sox, Gribbons [44], 2010	Yes	n.i.	n.a.	Before usability and performance testing, facilitators introduced the task using standardized scripts, explained the purpose of the reporting tool, and instructed participants how to complete the tasks.
35	Stewart, Letourneau [50], 2011	Yes	No	n.i.	n.i.
36	Swanson, Duijff [55], 2025	Yes	No	The intervention consisted of 8 weekly online sessions (2 h each), with practice exercises.	n.i.
37	Tanenbaum, Zaharieva [45], 2022	Yes	No	n.i.	n.i.
38	Thoren, Janson [64], 2021	Yes	n.i.	4 weekly group sessions followed by a 12-week web-based program.	n.i.
39	von Sengbusch, Doerdelmann [60], 2021	Yes	No	12 months	n.i.

### Theme 1: accessibility and usability

This theme captures parents´ needs and experiences regarding the practical use of the e-Health application, including ease of navigation, technical functionality, layout, and the effort required to access features and information. It focuses on how users access and interact with the e-Health application.

#### Needs

Parents prioritized anytime/anywhere access [61], including immediately after diagnosis and post-discharge from the hospital [33, 41]. They requested mobile-first design [27, 28], synchronization across platforms [37], minimal stream login barriers for low-stakes content [27, 28], directly accessible content [47], offline capability/low bandwidth functionality [48, 61], printable materials [29], and availability in multiple languages [48]. In terms of usability, parents preferred simple navigation [27, 52] and user-friendly and interactive interface [43, 53, 54]. They requested guided onboarding [44], drop down menus [44], engaging multimedia with a combination of text, images, asynchronous videos [27–29, 48, 57, 61], motivational messages [65], as well as appropriate incentives and gamification [28, 34, 37, 47, 48, 52, 57, 61, 65]. Moreover, push notifications were requested [27]. However, there was a strong emphasis on personalization, including adjustable home screen icons, contact frequency, content intensity, care preferences and goals with customizable templates and step-wise data entry [27, 29, 33, 44, 47, 57, 61].

#### Experiences

Parents generally found e-Health tools convenient, flexible, and easy to learn, particularly when available on smartphones and tablets, integrated smoothly into daily routines, and accessible immediately after diagnosis [28, 31, 32, 41, 49, 53, 54, 56, 59–62]. They valued time savings, home-based access to records and healthcare providers, and support while waitlisted [28, 32, 40, 61, 62, 64]. Short use times (5–15 min) were viewed as manageable [30, 62], while some tools were deemed as too time-intensive [53, 54]. Guided onboarding, personalization (e.g., goal setting/customization), and strategy videos improved engagement and learning [28, 43, 62]. E-health often increased parents’ sense of safety and was seen as relevant to family needs [53, 56, 62]. Nonetheless, unmet expectations and frustrations with usability were common. Barriers included limited time to use portals, forgotten or lost passwords, technical and connectivity issues, incompatible devices, and login/registration difficulties [28, 31, 32, 34, 43, 53, 54, 61]. Interface design was key: parents valued attractive layouts [27], customization, (e.g., personalized messages based on the health needs [43]), and clear navigation [27], but cluttered or stiff layouts, long introductions, repetitive or poor navigation, lack of subtitles, visually bland content, and limited customization options reduced usability [43, 54, 56, 65]. Excessive notifications further hindered use [54, 56]. Common technical issues included connectivity problems, syncing errors, software updates, and sound or video quality issues were also common [38, 39, 41, 49, 56, 59–61].

### Theme 2: content and information

This theme describes parents’ perceptions of the content provided by the e-Health application, including its relevance, completeness, and comprehensibility. It focuses on the type of information provided rather than how it is accessed in the above section.

#### Needs

Across conditions, concerns about misinformation, bias, and difficulty judging accuracy [27, 46, 51] highlighted the importance of evidence-based and accessible information. Families sought concise, trustworthy, and regularly updated guidance in plain language, supported by clear definitions, simple examples, diagrams, and bullet points [27, 29, 33, 44, 48, 51, 56, 57]. Families emphasized the need for a centralized hub consolidating credible resources, including reliable websites, local services, and regularly updated research [29, 42, 48, 57], supported by clearer communication from healthcare teams [51]. In terms of delivery of content, parents appreciated multimedia formats, such as short videos, step-by-step visuals, webinars, FAQs, and interactive downloadable materials [34, 57]. Once again, there was a strong emphasis on personalization concerning information provided to parents. Most parents requested condition-specific content. Specifically, core informational needs included the provision of explanations of diagnoses, causes, care pathways, treatment options, medications, side-effects, comorbidities, children’s quality of life, and tips for daily living with the chronic condition [29, 34, 57, 59, 65]. The delivery of culturally adapted materials was also regarded as essential [50, 51]. Notably, alongside reliable, research based information, it was important to parents to have access to adequately trained HCPs and experiential knowledge from peers [57].

#### Experiences

Parents´ experiences with the quality of the e-Health content were mixed but generally positive [27, 28, 32, 39–41, 55, 64]. They valued information that was relevant, trustworthy and clearly presented, with concise explanations on general care pathways, treatment options, coping strategies, and practical tips for daily living [27, 28, 32, 34, 35, 48, 55, 62, 65]. This broadly accessible guidance was seen as helpful especially early after diagnosis and was appreciated when presented in clear, concise, multimedia formats that supported comprehension and engagement [27, 40, 62]. However, parents reported the greatest benefit when content was condition-specific [34, 65]. Condition-specific resources enhanced confidence, coping, and decision making [34, 43, 53, 54, 62], while generic or overly technical information was often experienced as overwhelming, less relevant, or even anxiety-provoking [43–45, 48]. Parents felt it was difficult when the app was not specific to the child´s chronic condition [43]. Concerns regarding content persisted about accuracy, commercial bias, and difficulty judging personal relevance [31, 40, 44, 48]. Parents often highlighted feeling confused by difficult clinical terminology and concepts [40, 54], as well as information overload from long introductions and repetitive, non-engaging content [43, 54, 65]. Furthermore, parents noted that they sometimes saw information in the portal they wished they had not seen that frightened them, or which they would have preferred to receive directly from their provider [32].

### Theme 3: coordination (organization, monitoring, emergencies)

This theme reflects parents’ views on how e-Health applications could support the organization and coordination of care. It includes aspects, such as scheduling and tracking appointments, sharing relevant information, and facilitating continuity of care across different healthcare settings.

#### Needs

Parents stated that they would like e-Health to reduce organizational barriers – such as fragmented care, redundant paperwork, referral hurdles, long waiting times, and difficulties with scheduling and document management – by supporting coordination, collation, and communication with HCPs [29, 46, 57]. Specifically, parents requested easy online scheduling [31] and push notifications [27]. Another frequently requested feature was a central hub for care plans, meeting notes, symptom logs, lab results, school letters, online appointment booking, and customizable reminders for appointments, medications and check-ups [31, 34, 46, 47, 56], ideally with seamless multi-device synchronization [37, 59]. Parents also emphasized health data tracking, requesting for symptom/medication/activity diaries [29, 34, 65], progress monitoring (treatment and cognitive development) [29, 51], and single-point access with interoperability across devices [37, 59]. Additionally requested features included a family calendar for coordination [31], as well as emergency action plans, emotion logs, and automatic alerts to relatives and HCPs [34].

#### Experiences

Parents expressed that they valued e-Health for strengthening the organization and coordination of their child’s care through collated data hubs and digital communication features, which would increase confidence that their child’s condition was being properly managed [30, 54, 56]. Reported benefits included easier access to medical records [31], less complicated appointment scheduling [31, 48], and electronic prescriptions [31, 32]. Digital communication functions also supported more efficient information exchange – both between families and providers and across professionals within hospitals [31] – which improved care coordination and was experienced as reassuring [31, 32, 40, 59, 61]. However, some parents worried that providers might not review shared data consistently [30, 56] and preferred receiving some medical information directly from a clinic rather than via a portal [32]. Tracking and monitoring devices (e.g., wearables, sensor-based apps) were generally valued [34, 37, 56, 59]. One study found that 80% of parents considered them helpful, and 50% said they directly supported condition management [34]. These tools replaced paper-and-pencil records and consolidated data [37], increased situational awareness [63], supported routine formation [37], aided in decision making [63], and reduced parental worry [37, 58]. Parents appreciated a better overview of day-to-day situations [60] and valued predictive trend arrows and graphs [63]. Devices were also useful in emergencies, with alerts often seen as well-timed [37]. However, challenges included alarm fatigue found disruptive by children [58], data overload [45, 58], and setup/sync hurdles [37, 58, 63]. Moreover, continuous data sharing sometimes created family conflict, as children felt over-monitored and parents constant access to health and routine information reduced their sense of autonomy [58].

### Theme 4: consultation with clinicians

This theme encompasses parents’ perspectives on using the e-Health application to support their interactions with health care professionals (HCPs). It includes expectations regarding communication with HCPs, opportunities for remote consultation or follow-up.

#### Needs

Parents sought direct access and communication with medical specialists and HCPs [46, 57], conveying a strong preference for secure, two-way communication [28, 29, 31, 34, 56] through the means of asynchronous channels (messaging, video, email, SMS) [29, 34, 37, 56] and telephone [59] or video conferences [56]. Parents wanted expert hotlines for acute issues and easy sharing of plans with schools/other caregivers [59].

#### Experiences

Beyond better information sharing between parents and HCPs that improved organizational management, e-Health communication with providers, via chat functions or online consultations, consistently supported parents psychological well-being [36] and improved access, continuity, and confidence in exchanges with HCPs [32, 40]. Chat functions enabled frequent, ongoing dialogue that parents found reassuring [59]. However, usability issues hindered communication (non-consumer-friendly interfaces, platform clutter, difficult message deletion) [31, 61]. Yet, parents highlighted confidentiality/privacy and security concerns in chat environments [31, 47]. For online consultations, flexible scheduling and high visit frequency were helpful [59, 60]. Many parents preferred video over phone contact and often viewed it as comparable to in-person interaction [59, 60], with greater connectedness, more clinician “time”, and more focused dialogue that improved understanding [30, 59, 60]. Moreover, several parents believed that e-Health enhanced their providers ability to assess the child’s well-being [30]. However, some parents missed facial expressions and body language of HCPs and experienced weaker relationships via digital contact [49, 50]. Recurrent feedback across all parents was that they did not want face-to-face contact to be replaced [30, 56]. Additionally, internet instability/insufficient network, syncing issues, camera setup difficulties impeded conversations with HCPs [38, 41, 49, 59, 60].

### Theme 5: psychosocial support

This theme describes parents’ expectations regarding emotional and social support provided via the e-Health application. It includes features aimed at reducing stress and improving well-being, strengthening coping strategies, and facilitating peer exchange.

#### Needs

Parents noted that e-Health could support their psychosocial needs by connecting them with other affected parents, reducing loneliness through (positive) discussions, shared experiences and resource exchange [47, 50, 52, 56, 57, 61]. Interestingly, some parents preferred parent-to-parent advice over professional input [57]. They requested a messaging function in a potential app, as well as peer-to-peer communities [57], but noted that these communities should be non-judgmental spaces [27], with the ability to filter by condition/topic/location [58]. Most agreed these communities should be monitored, with clear rules for behavior and expert screening of contributions to ensure content accuracy [57], while some did not share these wishes [47]. Beyond peer exchange, parents also wanted psychological and emotional support for their children and themselves to reduce stress and improve mental health [57], as well as therapeutic and psychoeducational offers [52].

#### Experiences

Parents consistently valued opportunities to connect with other affect families for emotional support and practical advice [48, 55]. Online communities provided a supportive environment for exchanging struggles and successes, as well as practical tips regarding sleep, school, crises, and services [35]. Such group interactions, either via chat or video, reduced loneliness and enhanced coping, self-efficacy, and hope [36, 41, 48–50, 54]. Breakout rooms or personal chat functions were especially appreciated for fostering a greater sense of being understood and deeper connections [49]. Notably, anonymous or stimulating aspects of chat formats increased participation [50]. While some parents preferred receiving input from peers rather than from professionals [57], others expressed a preference for receiving certain information directly from clinicians [32]. Concerns included potential judgement, cyberbullying, misinformation and excessive screen time [48]. Beyond peer support, parents welcomed opportunities to attend to their own psychological needs (i.e., therapy or online coaching), which contributed to improved wellbeing [36, 52, 55]. Reflective and coping sessions were particularly valued [38], with parents reporting enhanced emotional functioning [48].

### Theme 6: data protection

This theme reflects parents’ views regarding the security and confidentiality of personal health information. It focuses on their concerns regarding, informed consent, privacy and trust in the e-Health application’s management of sensitive information, transparent data handling and user control over data sharing.

#### Needs

Families requested privacy, confidentiality, clear registration and consent as well as transparency about who can see what and the ability to control data sharing with clinicians, schools, and extended caregivers [47, 52].

#### Experiences

Parents´ experience with data protection using e-Health tools was mixed. While some reported no concerns regarding confidentiality and security of platforms [32, 49, 50], others expressed uncertainty about who could access their data and desired greater transparency in this regard [40]. Specific concerns about confidentiality, privacy and security were also raised in chatbot environments parents did not appreciate when the chatbot filtered or controlled access to information, as it created doubts about transparency [47]. Beyond data management, broader online safety risks, such as cyber-bullying and web-based predators, were noted, which sometimes undermined parental trust in digital platforms [48].

### Experiences and needs across the care pathway

Evidence on the timing of parental needs varied. While most studies did not specify care-trajectory stages [28, 30–37, 39–44, 46–55, 57, 58, 60, 61, 63], some addressed early phases (e.g., diagnosis) [38, 45, 59], transitional periods (e.g., hospital-to-home discharge) [29], or long-term management [27, 56, 62, 64, 65].

## Discussion

This scoping review systematically examined the needs and experiences of parents of children with chronic conditions regarding the use of e-Health applications. Previous reviews have predominantly been condition-specific [8, 17, 66], whereas this review is, to our knowledge, the first to employ a cross-diagnosis approach. By synthesizing evidence across diagnoses and modalities, the current review advances insights into the ways e-Health applications can align with parental needs and preferences while identifying critical research gaps.

The findings of this review highlight several key domains essential to the e-Health applications play in supporting parents of children with chronic conditions. Across studies, accessibility and usability were identified as fundamental requirements. Parents emphasized the importance of flexible, easily navigable, and time-efficient digital tools that can be accessed anywhere. Information quality was also central: parents valued resources grounded in up-to-date scientific evidence, presented in clear, non-technical language, and validated by experts. Support for family coordination of children’s disease management and patient journeys emerged as a major area for future action. Parents expressed the need for a unified platform to manage reminders, documents, symptom tracking, and medication schedules. Communication with HCPs was a strong parental priority; while parents appreciated the convenience of digital, two-way communication (e.g., via email, messaging, or video), they consistently underscored that such tools should complement rather than replace face-to-face interactions and should allow customization of frequency and mode. In addition, peer and psychosocial support were valued, with parents seeking secure, non-judgmental online spaces to connect with other parents in similar situations. Finally, data security and trust were recurring concerns, particularly regarding the use of chatbots and the protection of personal information. Collectively, these cross-diagnosis findings indicate that while certain needs-. especially those related to coordination - are common across conditions, parents consistently preferred tools and information that are personalized and diagnosis-specific, suggesting that e-Health applications should balance general functionality with tailored content to optimize engagement and effectiveness. Thus, the evidence points to the utility of universal design principles adaptable to condition-specific contexts.

### Interplay of experiences and needs

An overarching finding is the reciprocal relationship between parental needs and reported experiences. Needs for accessibility, reliable information, and support for care coordination were repeatedly reported by parents [31, 61]. Correspondingly, positive experiences were reported when applications addressed these domains - for example, through mobile apps supporting diseases management [34] or web-based portals facilitating access to health records and communication with providers [31, 61]. Where needs were unmet, such as limited customization, login friction, or lack if interoperability, parents reported frustration and disengagement [37, 54]. This underscores that unmet needs manifest directly as negative user experiences, highlighting the necessity of embedding parental priorities into all phases of design and evaluation.

### Temporal dimensions and the care trajectory

Our findings highlight an important area warranting further investigation: parental needs shift over the course of the child’s care trajectory. E-Health applications appeared especially valuable at the stage of the diagnosis, whereas providing orientation and reassurance [38, 59, 62] as well as supporting monitoring and coordination were more relevant during disease management longitudinally [27, 65]. Transitional phases, such as discharge from hospital to home, were less frequently topics of conversation in the included studies. However, other studies suggest a heightened need during these stages [29], showing that e-Health applications can support care coordination, social well-being, and emotional support for families of children with chronic conditions [67]. Findings suggest particular usefulness during early diagnosis and in long-term care, whereas transitional phases remain under-explored. This temporal pattern underscores the importance of adopting a longitudinal perspective when developing an evaluating e-Health applications, consistent with patient-journey frameworks in health service research. Importantly, our findings highlight that parental needs are not static but evolve over time, emphasizing the need for adaptable e-Health applications that can respond to changing requirements throughout the child’s care trajectory.

### Implications for theory

The findings of this review underscore the need to further investigate cross-diagnosis coordination needs among parents of children with chronic conditions. Coordination emerged as a universal challenge, suggesting that theoretical models of caregiving and digital health support should incorporate a cross-condition perspective. On the other hand, domains, such as information provision, communication, and psychosocial support consistently required diagnosis-specific tailoring. Future theoretical work should explore the type and degree of personalization required to adequately meet parents’ diverse needs. Evidence from other studies indicates that tailoring content, communication, and support to individual users is crucial for enhancing engagement and effectiveness, as personalization addresses users’ unique needs, preferences, and contexts [68]. Specifically, it is essential to determine how personalized features – such as goal setting, tailoring of information and messages to parents’ knowledge level, or to the severity of the child’s disorder – can enhance both perceived usefulness and engagement. Our findings also resonate with theories of technology acceptance, which emphasize perceived usefulness and trust in relational continuity as central determinants of technology adoption [69].

### Implications for practice

For health-care researchers and practitioners, the results support the development of a secure and trustworthy standardized e-Health application designed to assist parents with coordination tasks and psychosocial support across all conditions. Such a platform should function as a central hub; allowing parents to manage all relevant information, communication, and organizational activities in one place. While the application should be standardized to ensure accessibility and reliability, it should also allow for condition-specific modules that can be activated needed. These modules could provide tailored information about the child’s condition, enable communication with relevant professionals, and connect parents with peer support groups facing similar challenges. In this way, the system would combine general usability with personalized, diagnosis-specific relevance. These implications may also extend to other target populations. Adults living with (chronic) conditions or parents with children with acute conditions could similarly benefit from the advantages e-Health applications embedded in care. In addition, HCPs may benefit from streamlined communication and more efficient information exchange, which could facilitate care coordination and reduce administrative burden. As trusted sources of health information, HCPs may also serve as an important access point to e-Health interventions by introducing and recommending evidence-based applications to patients, thereby supporting their uptake and sustained use. These findings further highlight the importance of developing interoperable e-Health applications that are compatible with existing digital (and non-digital) health infrastructures and clinical workflows, thereby promoting seamless integration into routine care and maximizing benefits for both patients and HCPs.

For policymakers, the findings suggest that investment in digital health should not only prioritize technological innovation but also systematically incorporate parental feedback in design and evaluation. This observation is supported by previous studies [70]. Co-design approaches, which were followed in some included studies [45, 65], may be particularly effective in aligning tools with parental priorities. Embedding such participatory methods within policy and practice frameworks could enhance adoption, improve trust, and ensure that e-Health applications are responsive to the real-world needs of families. Integrating user-centered design principles represents an important strategy for the development and evaluation of future e-Health applications. By systematically involving end-users throughout iterative design cycles and applying heuristic evaluation methods, interfaces can be tailored to users’ workflows, cognitive models, and contextual needs, thereby improving efficiency, satisfaction, and safety. Future research and development efforts should therefore explicitly adopt user-centered methods and heuristic-based evaluations to further strengthen the usability, acceptability, and real-world effectiveness of e-Health applications [71–73].

Data security and trust also emerged as persistent barriers to adoption. Another source similarly highlighted the importance of implementing robust systems for data protection, legal compliance, informed consent, and the transition from paper-based to digital records to ensure confidentiality, data integrity, and user trust [26]. Even when the functionality of digital tools was appreciated, parents expressed hesitancy about data sharing and privacy protection. These concerns highlight that transparency, robust governance mechanisms, and institutional endorsement are not peripheral issues but essential design criteria. Aligning with broader digital health literature, trust must be considered a fundamental determinant of user engagement and sustained use. Digital health systems that prioritize transparency, clear communication about data use, and visible institutional accountability are more likely to achieve acceptance and long-term success among parent users. A comprehensive scoping review further confirms that institutional factors such as data protection, stakeholder engagement, strong governance, and transparency are central to building trust in digital health systems [74].

### Implications for public health

At a broader level, e-Health applications appear to be widely accepted among parents as a source of general support and stress reduction. Other studies have also found the potential to reduce caregiver stress [66]. Integrating e-Health applications into treatment plans for families of children with chronic conditions could therefore enhance well-being and reduce caregiver burden. Findings from a different study indicate that few interventions currently address caregivers’ mental health, highlighting the need for approaches that target both caregiving behaviors and caregivers’ well-being [66]. Public health strategies should allocate more resources toward optimizing and expanding both cross-diagnostic and diagnosis-specific e-Health applications. A combined model, offering universal features, such as coordination tools alongside condition-specific resources, may ultimately be the most efficient and desirable approach. It is central that the development of novel digital tools is grounded in a systematic identification of existing gaps in functionality, usability, accessibility, or integration with current digital or non-digital infrastructures in ongoing research. HCPs are uniquely positioned to detect these shortcomings, contribute to the design process, and align new solutions with established care pathways, thereby ensuring seamless interoperability and fostering rapid adoption across diverse clinical settings.

### Limitations and strengths

There are also limitations that should be considered when interpreting the findings. The included studies involved parents of children with a wide range of conditions, and experiences of parents may vary depending on the type and severity of the condition. However, these differences were not always clearly described. Moreover, chronic conditions were not consistently defined across the included studies, and the concept of e-Health was also used heterogeneously, which may limit the comparability of results. To address this issue, a pretest was conducted in which both screeners first applied and refined the inclusion and exclusion criteria to ensure clarity and relevance before the actual screening process began. Furthermore, the description of the study populations was often unclear in the included studies, making it difficult to assess the transferability of findings. The focus was on parents with chronically ill children in the age-group 0–16 years; all children or families that could not be clearly assigned to either the inclusion or exclusion criteria – for example, due to insufficient description of the study population – were excluded. Additionally, the review did not include grey literature, which may have led to the omission of relevant evidence. Articles published within the last 15 years and indexed in four databases were analyzed, which is in accordance with the JBI Scoping Review methodology. Finally, as quality assessment is not required in scoping reviews [20–22], hence, the methodological quality of the included studies was not evaluated.

This scoping review has several notable strengths. The inclusion and exclusion criteria were pretested to ensure their clarity, feasibility, and consistency, thereby enhancing the rigor of the study selection process. Furthermore, a comprehensive and well-structured search strategy was applied across multiple databases, increasing the likelihood of capturing a broad and representative range of relevant studies. A high level of inter-rater reliability was achieved during screening and data extraction, which strengthens the credibility and reproducibility of the findings. Collectively, these methodological strengths contribute to the robustness and transparency of the review and support the validity of its conclusions.

### Future research

In order to translate the identified evidence gaps into actionable research, we propose three concrete research questions and corresponding consecutive studies with varying study designs.

The following three research questions should be addressed in future research:

(1) “What are the quantitative and qualitative needs of parents caring for children with chronic conditions in the long term?”; (2) “Which Consolidated Framework for Implementation Research (CFIR)-derived determinants most strongly predict successful integration of new e-Health applications into routine care?”; and (3) “Does the combined delivery of a parent-centered e-Health application and provider training improve clinical outcomes compared with usual care or a waitlisted control group across different hospital settings and departments?” First, a mixed-methods cohort study should be conducted with parents of children living with chronic conditions to quantify unmet needs (e.g., access to disease-specific e-Health applications, digital health literacy) over the long term and to qualitatively explore situational barriers and facilitators they experience when interacting with e-Health applications. Second, an implementation-science investigation could apply the Consolidated Framework for Implementation Research (CFIR) to systematically assess the rollout of newly developed e-Health applications. In an implementation study, implementation indicators (fidelity, adoption, reach, sustainability), as well as indicators for stakeholder engagement and workflow integration would have to be pre-defined and contextual determinants that affect uptake would have to be explored. Third, a pragmatic multi-site-controlled trial could evaluate effectiveness: Participants would be randomized to (i) an intervention arm receiving the parent-centered e-Health application together with a structured training program for HCPs, (ii) an active control arm receiving usual care, and (iii) a waitlisted control arm receiving the e-health intervention after its completion in the first group. Randomization will be stratified by hospital and by department (e.g., pediatrics, endocrinology, pulmonology) to capture variability across chronic-condition services.

## Conclusions

This scoping review highlights the growing importance of e-Health applications in supporting parents of children with chronic conditions. While previous studies have shown that such tools can reduce parental stress and are generally perceived positively [11, 13, 51, 75], there has been limited evidence examining parental experiences across different diagnoses. By adopting a cross-diagnostic perspective, this review extends existing research beyond condition-specific insights and offers a more comprehensive understanding of parents’ needs, and experiences in digital health contexts.

The findings demonstrate that e-Health applications have considerable potential to strengthen parental support by improving coordination, facilitating timely assistance when access to healthcare is limited, and extending service reach – particularly in rural or underserved settings. However, to maximize their effectiveness, these tools should be both accessible and adaptable. E-Health applications should be designed in clear, non-technical language that matches parents’ health literacy and technological competence, and offer multilingual options to promote inclusivity. Additionally, compatibility across digital devices and transparent guidelines for peer communication are critical for maintaining trust, usability, and long-term engagement.

This review also emphasizes that while certain needs – such as coordination and data security – are shared across conditions, parents consistently desire diagnosis-specific personalization. Future research should therefore focus on determining the appropriate degree of customization and identifying the features that best address both common and unique parental needs. Ensuring that digital tools are trustworthy, secure, and integrated within existing care structures will be essential for their successful implementation.

## Supplementary Information

Additional file 1.

Additional file 2.

Additional file 3.

## Data Availability

All data generated or analyzed during this study are included in this published article and its supplementary information files.
